# Insulin-Like Growth Factor-I as an Effector Element of the Cytokine IL-4 in the Development of a *Leishmania major* Infection

**DOI:** 10.1155/2018/9787128

**Published:** 2018-07-29

**Authors:** Luiza C. Reis, Eduardo Milton Ramos-Sanchez, Fabricio Petitto-Assis, Audun H. Nerland, Maria Hernandez-Valladares, Frode Selheim, Lucile Maria Floeter-Winter, Hiro Goto

**Affiliations:** ^1^Instituto de Medicina Tropical de São Paulo, Universidade de São Paulo, IMTSP-USP, São Paulo, SP, Brazil; ^2^Department of Clinical Science, University of Bergen, Bergen, Norway; ^3^Departament of Biomedicine, University of Bergen, Bergen, Norway; ^4^Departamento de Fisiologia, Instituto de Biociências, Universidade de São Paulo, São Paulo, SP, Brazil; ^5^Departamento de Medicina Preventiva, Faculdade de Medicina, Universidade de São Paulo, São Paulo, SP, Brazil

## Abstract

Certain cytokines modulate the expression of insulin-like growth factor- (IGF-) I. Since IL-4 and IGF-I promote growth of the protozoan *Leishmania major*, we here addressed their interaction in downregulating the expression of *Igf-I* mRNA using small interfering RNA (siRNA) in *Leishmania major*-infected macrophages. Parasitism was decreased in the siRNA-treated cells compared with the nontreated cells, reversed by the addition of recombinant IGF-I (rIGF-I). In IL-4-stimulated macrophages, parasitism and the *Igf-I* mRNA amount were increased, and the effects were nullified upon siRNA transfection. IGF-I downregulation inhibited both parasite and macrophage arginase activation even in IL-4-stimulated cells. Searching for intracellular signaling components shared by IL-4 and IGF-I, upon siRNA transfection, phosphorylated p44, p38, and Akt proteins were decreased, affecting the phosphatidylinositol-3-kinase (PI3K)/Akt pathway. In *L. major*-infected C57BL6-resistant mice, the preincubation of the parasite with rIGF-I changed the infection profile to be similar to that of susceptible mice. We conclude that IGF-I constitutes an effector element of IL-4 involving the PI3K/Akt pathway during *L. major* infection.

## 1. Introduction

Certain physiological processes are reciprocally controlled by the immune and endocrine systems, where the actions of cytokines and hormones efficiently regulate these processes [1–3]. In this context, the observation that certain cytokines modulate the expression of the hormone insulin-like growth factor- (IGF-) I [4–6] led us to explore this cross talk in the interaction of the *Leishmania* parasite with host cells since the essential roles of different cytokines and the effect of IGF-I on the development of *Leishmania* infection are known [7].

*Leishmania* is an obligatory intracellular protozoan that is transmitted vectorially and causes diseases called leishmaniases that affect two million people globally each year. These infections result in lesions on the skin, mucosa, or viscera, depending on the parasite species and the host response and features [8].

*Leishmania* infection leads to specific activation of the host cellular immune response, in which macrophages that harbor the parasite play a fundamental role in infection progression. Cytokines and growth factors, including IGF-I, act on macrophage leishmanicidal or parasite growth-promoting mechanisms. The metabolic products of L-arginine exert primary roles in these processes. Nitric oxide (NO), a main leishmanicidal element, is generated through the oxidation of L-arginine catalyzed by the inducible nitric oxide synthase (NOS2). In contrast, polyamines are essential nutrients for the growth of *Leishmania*, which are generated through the hydrolysis of L-arginine by arginase [9–11].

We began studying the role of IGF-I in *Leishmania* infection based on the observation that IGF-I is produced by different cell types, including macrophages, and is present in the skin, which is the tissue where the parasite initiates infection [12]. The addition of extrinsic recombinant IGF-I to cultures at a physiological concentration induces the proliferation of various species of *Leishmania* promastigotes and axenic amastigotes [13–15], promotes an increase in macrophage arginase-1 (ARG1) expression as well as in *Leishmania* arginase (*Larg*), *in vitro* [16, 17], and induces a significant increase in the lesion size and the number of viable parasites at the lesion site in experimental models [18]. Furthermore, macrophages contain endogenous IGF-I in the cytoplasm, which interacts with intracellular *Leishmania* parasites [19].

The initial data on the adaptive immune response in different mouse strains infected with *L. major* have shown that the susceptibility or resistance to this infection is related to the production of certain cytokines of the Th1 or Th2 profile, respectively, IFN-*γ*, or IL-4 and IL-13 [20–24]. Nevertheless, studies have indicated changes in the simplicity of this model with time. The production of IL-4 in resistant mice does not alter the evolution towards progressive disease, as expected. C3H mice that were treated with IL-4 or anti-IL-12 early in the infection developed a strong but transient increase in IL-4 level, with no change in their resistant phenotype [25–27]. Furthermore, the transfer of BALB/c cells with the high expression of IL-4 to genetically resistant chimeric mice on a C57BL/6 background did not result in susceptibility [28]. The findings from these studies thus suggest that the profiles of resistance and susceptibility are not exclusively due to Th1 and Th2 cytokines. Considering our previous results revealing an important role for IGF-I in the development of *Leishmania* infections and considering the differences in IGF-I expression in different strains of mice [29], IGF-I may emerge as a factor that could explain these differences. Furthermore, IFN-*γ* stimulation decreases IGF-I levels, whereas IL-4 and IL-13 stimulation increase IGF-I expression [4–6].

In the present work, we evaluated the role of intrinsic IGF-I in *L. major-*infected macrophages *in vitro* and its participation in modulating the effects of cytokines by silencing the expression of the *Igf-I* mRNA using a small interfering RNA. We studied the effects on parasitism and the L-arginine pathway upon cytokine stimulation. We next evaluated the effect on lesion development upon the injection of *L. major* promastigotes preincubated with recombinant IGF-I (rIGF-I) into the footpads of BALB/c and C57BL/6 mice, which differ in basal *Igf*-I mRNA amount.

Focusing on immune-endocrine cross talk, we are the first to observe a process in which a growth factor (i.e., IGF-I) acts as an effector element for the cytokine IL-4 in the induction of susceptibility to *L. major* infection since the expected parasite growth upon IL-4 stimulation was halted by silencing the IGF-I mRNA. The mechanism was directly related to the expression and activation of arginase and by the activation of the phosphatidylinositol-3-kinase (PI3K)/Akt pathway in macrophages. Furthermore, the injection of *L. major* promastigotes preincubated with rIGF-I into resistant C57BL/6 mice rendered the animals susceptible to the infection. The data thus suggest an essential role for IGF-I in the effect of IL-4 on the alternative macrophage activation pathway during *L. major* infection.

## 2. Materials and Methods

### 2.1. Mice

Six- to 8-week-old inbred BALB/c and C57BL/6 mice were obtained from the Animal Facility of the Faculdade de Medicina, Universidade de São Paulo (USP), and maintained in the animal facility of the Instituto de Medicina Tropical de São Paulo, USP.

### 2.2. Parasites

*L. major* LV39 (MRHO/Su/59/P) was maintained through regular passaging in BALB/c mice, and promastigotes were derived from amastigotes purified from the footpad lesions of BALB/c mice and expanded and maintained in 199 medium (Cultilab, Brazil) supplemented with 10% heat-inactivated fetal calf serum (FCS) (Cultilab, Brazil) at 26°C. The promastigotes used in the experiments were in the stationary phase of growth and had undergone no more than four passages in culture. All procedures were approved by the institutional guidelines for animal care and use and by the ethics committee of the institution.

### 2.3. Evaluation of the Effect of IGF-I on Promastigotes in Culture

*L. major* promastigote cultures (5 × 10^5^/mL) were established in 24-well plates (Corning Costar, USA) in 199 medium (Cultilab, Brazil) supplemented with 2% heat-inactivated fetal calf serum (FCS) (Cultilab, Brazil) at 26°C, and parasites were stimulated with or without 50 ng/mL IGF-I (R&D Systems, USA). Parasites were counted daily under a light microscope (Carl Zeiss, Gottingen, Germany), and the result was expressed as the number of parasites × 10^7^/mL followed for 10 days of culture.

### 2.4. Infection of Macrophages with *L. major*

The RAW 264.7 macrophage cell line (ATCC) and BALB/c and C57BL/6 peritoneal macrophages were grown in Dulbecco's modified Eagle's medium (DMEM) supplemented with 0.5% bovine serum albumin (BSA, insulin-free, Sigma, USA). Cells (5 × 10^5^ or 2 × 10^6^) were dispensed onto round 13 mm^2^ glass cover slips, which were placed in the wells of 24-well plates (Corning Costar, USA) and incubated for 30 minutes at 37°C in a humid atmosphere with 5% CO_2_ to allow adhesion. Thereafter, the wells were washed twice with culture medium to remove nonadherent cells. Then, the amastigote or promastigote suspensions (eight parasites per cell) were dispensed into the wells, and infection was allowed to occur for 4 hours at 33°C in a humid atmosphere with 5% CO_2_. After incubation, the noninternalized parasites were washed away. In one set of experiments, macrophages were treated with the Th1 cytokine IFN-*γ* (200 U/mL) or the Th2 cytokines IL-4 (2 ng/mL) and IL-13 (5 ng/mL), both separately and in combination. In other experiments, recombinant IGF-I (50 ng/mL; rIGF-I, R&D Systems, Minneapolis, MN, USA) was added. The culture was then maintained for 48 hours at 37°C in a humid atmosphere with 5% CO_2_.

### 2.5. Parasite Load in Macrophages

Cover slips were removed from the plates, and the slides were stained with Giemsa dyes, mounted, and processed to evaluate parasitism under a light microscope (Carl Zeiss, Germany). 600 cells per group were counted. The data are presented as the number of parasites per 100 cells [(number of parasites/number of infected cells) × (number of infected cells/total number of cells) × 100]. The analysis was performed by two independent observers who were blinded to the experimental conditions.

### 2.6. NO Production

Nitrite (NO_2_) accumulation in the cell culture supernatants was used as an indicator of NO production and was determined using the Griess reaction [30]. Fifty microliters of the culture supernatant was reacted with 50 *μ*L of Griess reagent (1% sulfanilamide, 0.1% N-(1-naphthyl)ethylenediamine dihydrochloride, and 2.5% phosphoric acid in bidistilled water) for 10 min at room temperature. The absorbance was measured at 540 nm using a Multiskan MCC/340 P version 2.20 plate reader (Labsystems), and the nitrite concentration was calculated using a standard curve for sodium nitrite (NaNO_2_). The tests were run in triplicate.

### 2.7. Arginase Activity

Cells and infected cells were removed from the culture, lysed, and used to determine arginase activity [31]. Briefly, 50 *μ*L of lysates was treated with the same volume of 10 mM MnCl_2_ and 50 mM Tris-HCl, pH of 7.4 at 56°C for 10 min to activate arginase. Then, 25 *μ*L of 0.5 M L-arginine at a pH of 9.7 was added to 25 *μ*L of the activated lysate and incubated at 37°C for 60 min. The reaction was stopped with 400 *μ*L of H_2_SO_4_/H_3_PO_4_/H_2_O (1/3/7, *v*/*v*/*v*). The urea concentration was measured at 540 nm using a spectrophotometer Multiskan MCC/340 P version 2.20 plate reader (Labsystems, Vantaa, Finland) after the addition of 25 *μ*L of 9% *α*-isonitrosopropiophenone in 100% methanol and incubation 100°C for 45 min. One unit of enzyme activity is defined as the amount of enzyme that catalyzes the formation of 1 *μ*mol of urea per minute.

### 2.8. RNA Extraction and Reverse Transcription

Total RNA was extracted from 2 × 10^6^ cells using TRIzol (Invitrogen, USA), according to the manufacturer's protocol. RNA integrity was verified by agarose gel electrophoresis/ethidium bromide staining and spectrophotometry (OD260/280 absorption ratio greater than 1.8). Total RNA (1 *μ*g) was reverse transcribed using the High Capacity cDNA Reverse Transcription kit (Applied Biosystems, USA) following the manufacturer's protocol and then stored at −20°C.

### 2.9. Quantitative qPCR

For real-time quantitative qPCR, we used the corresponding primer pairs for mouse sequences (*Igf-I*: 5′ TAC TTC AAC AAG CCC ACA GG 3′ and 5′ AGT CTT GGG CAT GTC AGT GT 3′; *Arg1*: 5′ AGC ACT GAG GAA AGC TGG TC 3′ and 5′ CAG ACC GTG GGT TCT TCA CA 3′; *Larg*: 5′ CAA CAC CAT GTC TGG TAC GGT CTC 3′ and 5′ CAC AGC ACG TAG ACC AAT GTA GGC 3′; *Nos2*: 5′ AGA GCC ACA GTC CTC TTT GC 3′ and 5′ GCT CCT CTT CCA AGG TGC TT 3′; *Cat-2b*: 5′ TAT GTT GTC TCG GCA GGC TC 3′ and 5′GAA AAG CAA CCC ATC CTC CG 3′; *β-actin*: 5′ GCC TTC CTT CTT GGG TAT GGA ATC 3′ and 5′ ACG GAT GTC AAC GTC ACA CTT CAT 3′; and *GAPDH*: 5′ AAC GAG AAG TTC GGC ATA GTC GAG 3′ and 5′ ACT ATC CAC CGT CTT CTG CTT TGC 3′). Reactions including master mix (SYBR® Green; Applied Biosystems, USA), 0.3 *μ*M of each primer, and 1 *μ*g of cDNA templates were run in triplicate on a PCR system (StepOne; Applied Biosystems, USA). The PCR conditions were the same for all primer combinations: 95°C for 10 min and 40 cycles of 92°C for 2 min, 57.5°C for 30 s, and 70°C for 30 s. After amplification, a melting curve was used to confirm the specificity of the target product. The relative expression data were quantified using the 2^−*Δ*ΔCt^ method [32].

### 2.10. siRNA

Small interfering RNAs (siRNAs) targeting murine IGF-I were designed using GenBank (NM_010512) and IDT SciTools RNAi Design (Integrated DNA Technologies) (*sense* 5′ AAA GGA GAA GGA AAG GAA GUA CAT T 3′ and *antisense* 5′AAU GUA CUU CCU UUC CUU CUC CUU U 3′). Universal scrambled siRNAs were employed as controls (Invitrogen, USA). Macrophages were grown in DMEM supplemented with 0.5% bovine serum albumin (insulin-free BSA, Sigma, USA) in a 24-well plate and were transiently transfected with a 150 *μ*M siRNA duplexes using Lipofectamine™ 2000, according to the manufacturer's instructions (Invitrogen, Carlsbad, CA, USA). Cells transfected with IGF-I siRNA were used after 48 h. The transfection efficiency was confirmed by real-time PCR and confocal microscopy. The inhibition percentage (% KD) for the IGF-I mRNA was calculated using the formula% KD = ([1 − 2^−ΔΔCt^] × 100) [32].

### 2.11. Confocal Microscopy

For immunofluorescence staining, cells infected with *L. major* promastigotes were grown on glass slides. Twenty-four hours after infection, cells were fixed with 4% paraformaldehyde, washed with 0.001 M phosphate-buffered saline (PBS; pH 7.2), blocked for one hour with 2% BSA/PBS, and then incubated overnight with a monoclonal goat anti-mouse IGF-I antibody (1 : 75, R&D Systems, USA) and a polyclonal mouse anti-*Leishmania* antibody (1 : 400) produced in our laboratory. Alex Fluor 546-conjugated donkey anti-goat IgG (1 : 200, Invitrogen, USA) and Alexa Fluor 488-conjugated donkey anti-mouse IgG (1 : 400, Invitrogen, USA) were used as secondary antibodies. Fluorescence imaging analyses were performed using a Zeiss LSM 510 Meta laser-scanning confocal microscope. As a negative control, the primary antibodies were omitted from the reaction.

### 2.12. SDS-PAGE and Western Blot

Cell lysates (20 *μ*g of protein in 20 *μ*L) were separated on a denaturing gradient 4–12% Bis-Tris NuPAGE gel (Invitrogen, USA) according to the manufacturer's instructions. Briefly, the separated proteins were blotted onto iBlot™ 2 Transfer Stack PVDF mini-membranes using an iBlot Dry Blotting System (Invitrogen, USA). Membranes were blocked with TBS-T buffer (150 mM NaCl, 20 mM Tris, 0.01% Tween 20, pH of 7.4) containing 5% fat-free milk for 1 h. Membranes were reacted with a 1 : 2000 dilution of a primary anti-phospho-Akt (Ser473) antibody, a 1 : 2000 dilution of a rabbit anti-p44/42 MAPK (137F5) mAb, and a 1 : 2000 dilution of a rabbit anti-p38 MAPK (D13E1) XP® mAb (Cell Signaling Technology, USA) overnight at 4°C and incubated with 1 : 1000 dilutions of a peroxidase-conjugated polyclonal anti-rabbit IgG and HRP-conjugated anti-biotin antibody (Cell Signaling Technology, USA) for 1 h at room temperature. Biotinylated protein molecular weight markers (Cell Signaling Technology, USA) were used. Bound antibodies were detected with an ECL chemiluminescence kit LumiGLO® Reagent (Cell Signaling Technology, USA) according to the manufacturer's instructions. An evaluation of the phosphorylation kinetics was conducted at different time points (0, 5, 10, 20, 30, and 60 minutes) after adding the stimulus (data not shown), and we chose the time of 30 minutes to present the results. Subsequently, protein bands were quantified by densitometry using AlphaEaseFC™ software 3.2 beta version (Alpha Innotech Corporation, San Leandro, CA, USA), and the results are expressed in arbitrary units, which were calculated by integrating the intensity of each pixel over the spot area and normalizing to the gel background.

### 2.13. Development of Footpad Lesions in Mice

Stationary-phase promastigotes (1 × 10^6^) that had been preincubated with or without 50 ng/mL of recombinant human IGF-I (R&D Systems, USA) for 5 min and then washed were injected into the footpads of BALB/c and C57BL/6 mice. In the opposite footpad, we injected RPMI 1640 medium as a control. For six weeks, we measured the thickness of the foot to indicate the growth of the lesions using a micrometer (Mitsutoyo, Japan), and the difference between the infected and noninfected footpads in millimeters was considered the lesion size.

### 2.14. Statistical Analysis

Statistical analyses were performed with GraphPad Prism 5 software (GraphPad Software Inc., San Diego, CA, USA). Data were subjected to analysis of variance (ANOVA) and Tukey's posttest and were considered significant when *P* < 0.05.

## 3. Results

### 3.1. The Effect of IGF-I on *L. major* in Culture and within Macrophages

**Figure 1:**
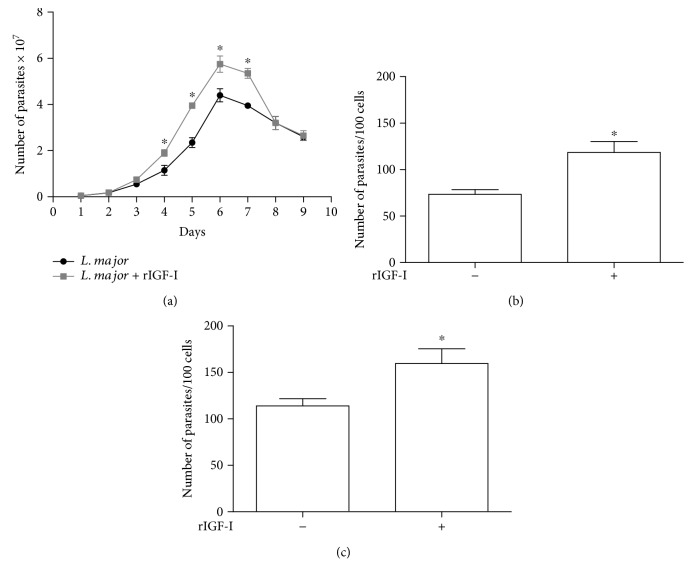
The effects of IGF-I on the growth of *L. major* in culture and within macrophages (mean ± standard deviation). (a) The effect of IGF-I on the growth of *Leishmania* promastigotes in culture maintained in 199 medium supplemented with 2% heat-inactivated FCS at 26°C is shown. *Leishmania* promastigotes were maintained with (gray line) or without (black line) 50 ng/mL rIGF-I. (b and c) The effect of IGF-I on the growth of *Leishmania* within macrophages is shown. RAW 264.7 cells were infected with *L. major* promastigotes (b) or amastigotes (c) treated with or without 50 ng/mL rIGF-I and incubated for 48 h. ^∗^*P* < 0.05 (one-way ANOVA and Student's *t* test) compared to the control without rIGF-I.

We first verified the effect of IGF-I on parasite growth *in vitro*. In the cultures stimulated with rIGF-I, we observed a larger number of parasites that reached the stationary phase earlier than nonstimulated cultures (Figure 1(a)).

When we evaluated the effect of IGF-I on the parasite burden in *L. major* promastigote- or amastigote-infected macrophages, we observed a significant increase in the number of parasites in macrophages stimulated with rIGF-I compared with that in nonstimulated macrophages (Figure 1(b)). Similar results were observed when amastigotes were used (Figure 1(c)).

### 3.2. The Effects of *Leishmania* Infection and Th1 and Th2 Cytokines on IGF-I Expression

**Figure 2:**
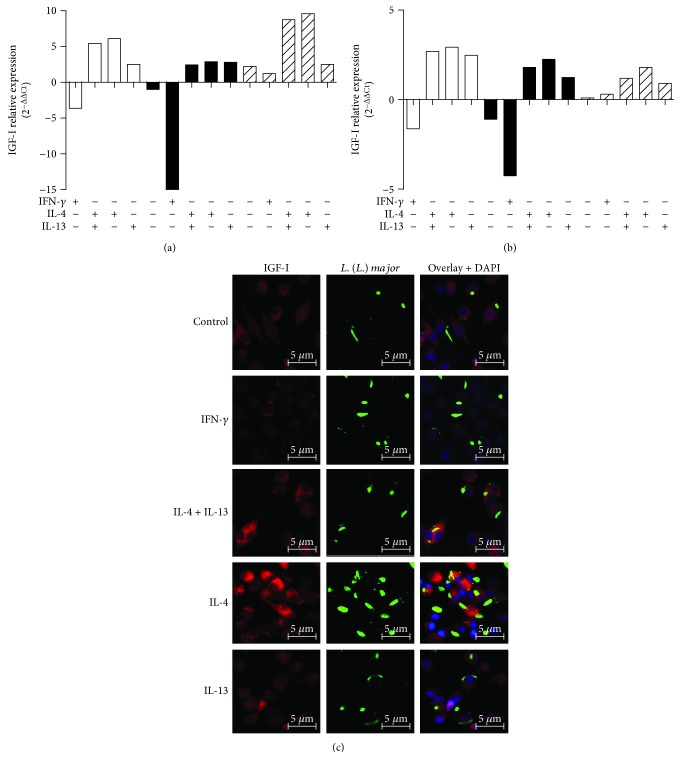
IGF-I expression in response to cytokine treatment. (a and b) Quantification of *Igf-I* mRNA in *L. major* promastigote- (black bars) or amastigote-infected (striped bars) or noninfected (white bars) RAW 264.7 cells (a) and BALB/c peritoneal macrophages (b) is shown. Cells were stimulated with IFN-*γ* (200 U/mL), IL-4 (2 ng/mL), and IL-13 (5 ng/mL) for 48 hours. One representative experiment from three independent assays is shown. (c) Detection of IGF-I expression using confocal microscopy of cells labeled with a 1 : 75 dilution of an anti-IGF-I antibody (using an Alexa Fluor 546-conjugated secondary antibody; red) and a 1 : 200 dilution of an anti-*Leishmania* antibody (using an Alexa Fluor 488-conjugated secondary antibody; green) in *L. major* promastigote-infected macrophages is shown. Nuclei were stained with DAPI (blue). Images were captured using a confocal Leica LSM510 microscope with a 63x oil immersion objective. The expressions are relative to the expression in untreated cells (the baseline).

We analyzed the effects of *Leishmania* infection and Th1 and Th2 cytokines on the amount of the *Igf-I* mRNA. Infection of RAW 264.7 cells and BALB/c mouse peritoneal macrophages with promastigotes decreased the amount of *Igf-I* mRNA compared with that of the uninfected control cells. When promastigote-infected RAW 264.7 cells were stimulated with IFN-*γ*, we observed a 15-fold decrease in the amount of *Igf-I* mRNA. When cells were simultaneously stimulated with IL-4 and IL-13, we observed a 2.45-fold increase in the amount of *Igf-I* mRNA compared to that in the nonstimulated controls. When cells were stimulated with either IL-4 or IL-13, we observed a 2.9- or 4.8-fold increase in the amount of *Igf-I* mRNA, respectively (Figure 2(a)).

Upon amastigote infection of RAW 264.7 cells, we observed different amounts of *Igf-I* mRNA that increased in all infected cells compared with that in uninfected controls. A 2.2-fold increase in the amount of *Igf-I* mRNA was observed upon amastigote infection, a 1.2-fold increase was observed following stimulation with IFN-*γ*, and an 8.7-fold increase was observed when cells were simultaneously stimulated with IL-4 and IL-13. When cells were stimulated with either IL-4 or IL-13, we observed a 9.2- or 2.5-fold increase in the amount of *Igf-I* mRNA, respectively (Figure 2(a)). A similar profile was observed in BALB/c peritoneal macrophages (Figure 2(b)).

We confirmed these results using confocal microscopy. In *Leishmania* promastigote-infected macrophages, we observed a decrease in IGF-I immunostaining upon stimulation with IFN-*γ* compared with that in control cells. When cells were simultaneously stimulated with IL-4 and IL-13 or with IL-4 alone, we observed an increase in immunostaining, indicating increased IGF-I expression (Figure 2(c)).

### 3.3. Inhibition of IGF-I Expression Using an IGF-I siRNA

**Figure 3:**
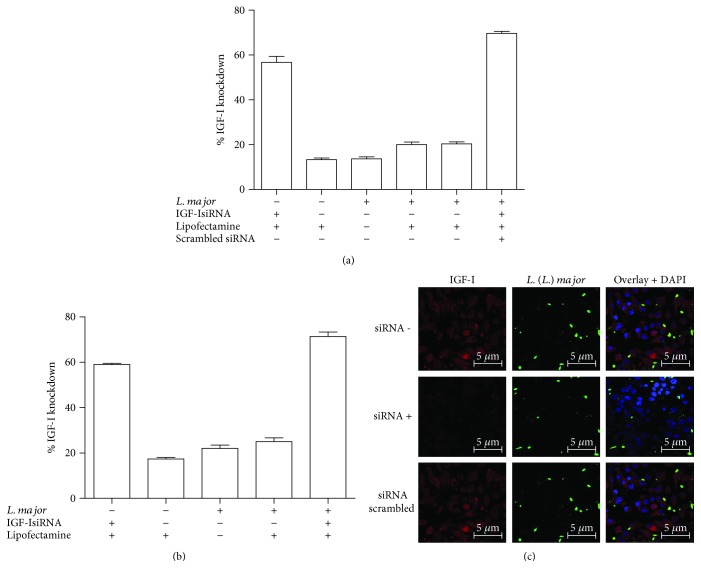
Expression of *Igf-I* mRNA upon IGF-I silencing with an siRNA. The percentage decrease in the amount of *Igf-I* mRNA in RAW 264.7 cells (a) or BALB/c peritoneal macrophages (b) infected with *L. major* promastigotes that were transfected with 150 *μ*M siRNA, scrambled siRNA, or Lipofectamine alone 6 h after infection is shown. One representative experiment from three independent assays is shown. (c) The detection of IGF-I expression in *L. major* promastigote-infected RAW 264.7 cells transfected with (siRNA+) or without the IGF-I siRNA (siRNA−) or with a scrambled siRNA using confocal microscopy of immunostaining with a 1 : 75 dilution of an anti-IGF-I antibody (using an Alexa Fluor 546-conjugated secondary antibody; red) and a 1 : 200 dilution of an anti-*Leishmania* antibody (using an Alexa Fluor 488-conjugated secondary antibody; green) is shown. Nuclei were stained with DAPI (blue). Images were captured using a confocal Leica LSM510 microscope with a 63x oil immersion objective.

Silencing IGF-I expression with siRNA resulted in an approximate 70% decrease in the mRNA amount in promastigote-infected RAW 264.7 cells (Figure 3(a)) and 78% in infected BALB/c peritoneal macrophages (Figure 3(b)). Using confocal microscopy, we observed a significant reduction in IGF-I immunostaining after siRNA transfection. Moreover, infection with *L. major* promastigotes alone inhibited IGF-I expression. A nonspecific double-stranded RNA (scrambled siRNA) was used as a negative control (Figure 3(c)). We observed similar results in cells infected with amastigotes (data not shown).

### 3.4. The Effect of IGF-I siRNA on Parasitism

**Figure 4:**
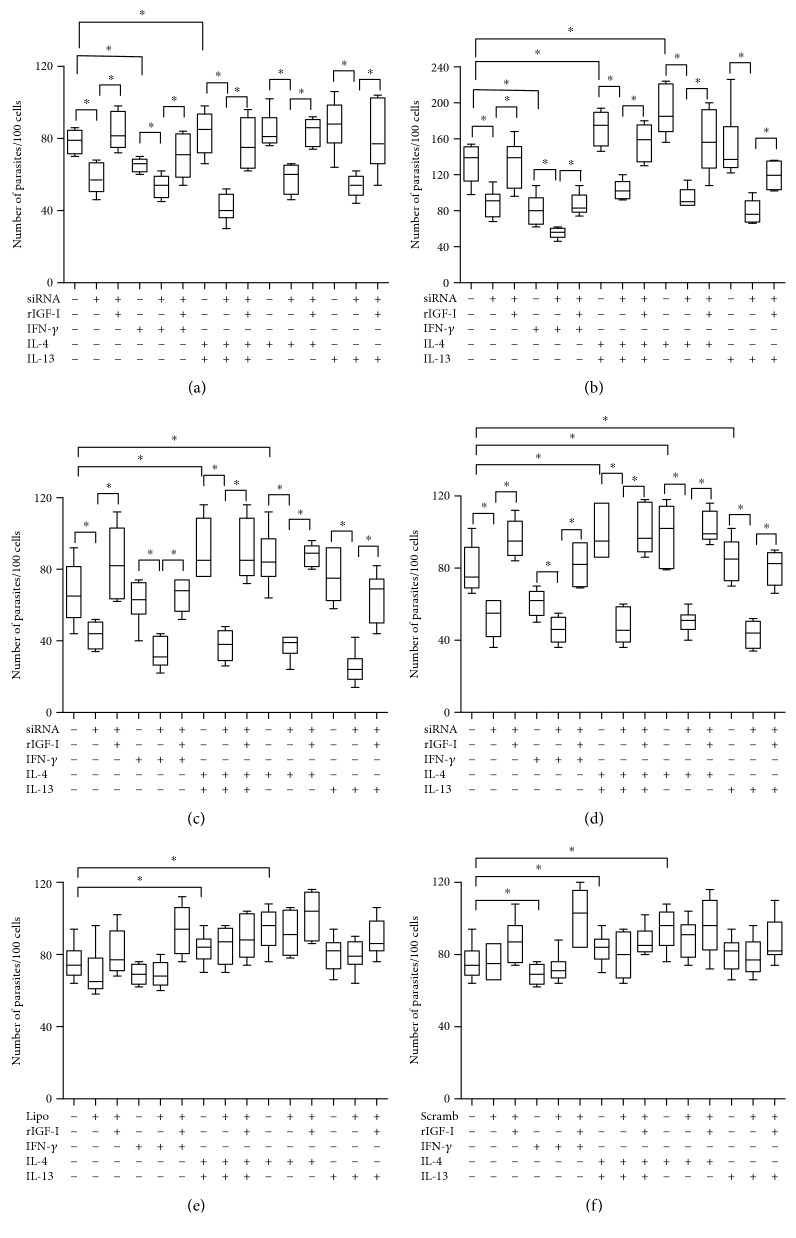
Parasitism in response to cytokine treatments and siRNA transfection. Parasitism (median number of parasites per 100 cells) in *L. major*-infected RAW 264.7 cells (a, b, e, and f) or BALB/c peritoneal macrophages (c and d) following transfection with IGF-I siRNA (a, b, c, and d), Lipofectamine alone (e), or scrambled siRNA (f) along with cytokine stimulation is shown. Promastigote- (a, c, e, and f) or amastigote-infected (b and d) cells transfected with or without siRNA or Lipofectamine were stimulated with IFN-*γ* (200 U/mL), IL-4 (2 ng/mL), IL-13 (5 ng/mL), and recombinant IGF-I (rIGF-I, 50 ng/mL) for 48 hours. One representative experiment from three independent assays is shown. ^∗^*P* < 0.05 (ANOVA and Tukey's tests).

Following infection with both promastigotes and amastigotes, we observed a significant increase in parasitism in cells exposed to Th2 cytokines (*P* < 0.05), as expected. In Th1 cytokine-stimulated cells, we noted a significant reduction in parasitism compared with that in the respective control (*P* < 0.05) (Figure 4).

Upon silencing IGF-I expression with an siRNA in *L. major* promastigote-infected RAW 264.7 cells, we observed a decrease in the parasite number from 79 parasites (median number in the control) to 57 parasites per 100 cells. In amastigote-infected cells, the parasite number was reduced from 139 to 91 parasites per 100 cells (*P* < 0.05). When we analyzed the involvement of Th1 and Th2 cytokines in parasitism following IGF-I silencing, all cells transfected with the siRNA showed a significant decrease in parasitism compared with that in the controls lacking siRNA following infection with both promastigotes and amastigotes (Figures 4(a) and 4(b)). The parasite load did not increase, even in Th2 cytokine-stimulated cells.

We observed similar results in *L. major*-infected BALB/c peritoneal macrophages to those obtained in *L. major*-infected RAW 264.7 cells, reinforcing the findings obtained from the RAW 264.7 cells (Figures 4(c) and 4(d)).

Experiments were performed to restore IGF-I activity after knockdown to ascertain the role of IGF-I. After transfection with siRNA, rIGF-I (50 ng/mL) was added to replace the loss of intracellular IGF-I, followed by an evaluation of parasitism. After the addition of rIGF-I to all cells transfected with siRNA, parasite numbers increased to the levels similar to the control cells lacking siRNA (Figure 4). We did not observe differences in parasitism in control cells treated with Lipofectamine or transfected with the scrambled siRNA compared with that in control cells. Infections with amastigotes or promastigotes produced similar results (Figures 4(e) and 4(f)).

### 3.5. The Effects of IGF-I siRNA on the mRNA Amount of *Nos2*, Arginase (*Arg1*), and Cationic Amino Acid Transporter 2 (*Cat-2B*) in Macrophages and *Leishmania* Arginase (*Larg*) mRNA Expression and Enzyme Activity

**Figure 5:**
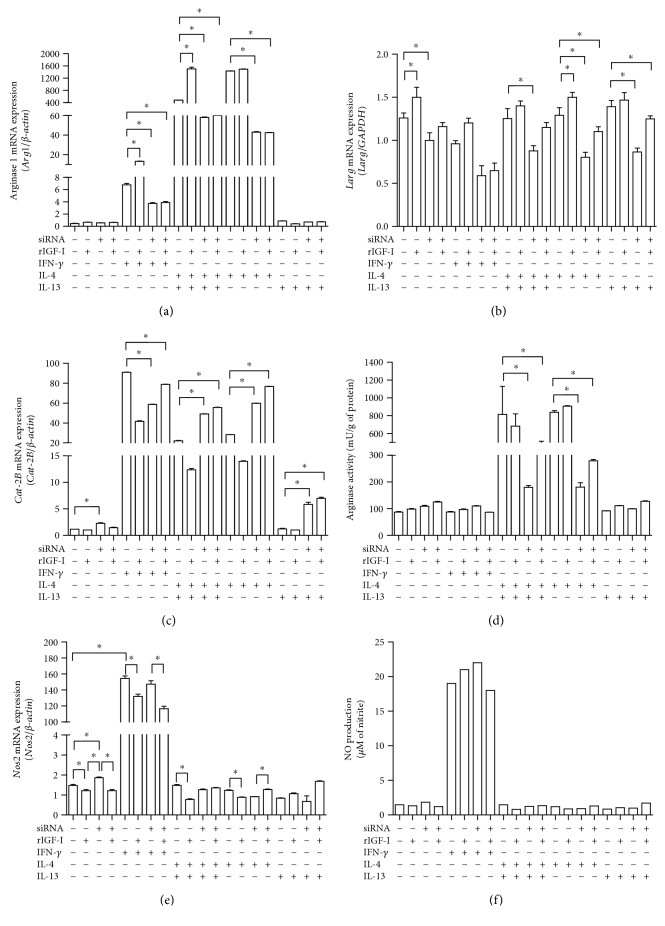
The effects of IGF-I siRNA transfection on the L-arginine metabolic pathway. The quantification of (a) *Arg1* mRNA, (b) *Larg* mRNA, (c) *Cat-2B* mRNA, (d) arginase activity, (e) *Nos2* mRNA, and (f) NO production in *L. major*-infected RAW 264.7 cells following transfection with IGF-I siRNA and cytokine stimulation is shown. Promastigote-infected cells transfected with or without IGF-I siRNA were stimulated with IFN-*γ* (200 U/mL), IL-4 (2 ng/mL), and IL-13 (5 ng/mL) for 48 hours. One representative experiment from three independent assays is shown. ^∗^*P* < 0.05 (ANOVA and Tukey's tests) compared to the respective control without siRNA.

We analyzed the effects of IFN-*γ* and IL-4 and/or IL-13 in combination with levels of IGF-I on L-arginine metabolism. IL-4 stimulation produced a significant increase in both *Arg1* (Figure 5(a)) and *Larg* mRNAs (Figure 5(b)), as well as an increase in arginase activity (Figure 5(d)). However, IL-13 stimulation did not show an increase in *Arg1* mRNA or arginase activity (Figures 5(a) and 5(d)) but showed an increase in *Larg* mRNA (Figure 5(b)). A significant increase in the amount of *Cat-2B* mRNA (Figure 5(c)) was observed after treatment with IL-4 (*P* < 0.05) compared to that in the control group.

When cells were treated with siRNA, all groups showed a significant decrease in both the *Arg1* and *Larg* mRNA amounts and arginase activity and an increase in the *Cat-2B* mRNA amount (Figure 5(c)).

When IGF-I was restored by adding rIGF-I to cells transfected with siRNA, we observed increases in both the *Larg* mRNA amount and arginase activity. No differences in the *Arg1* mRNA amount were observed in macrophages.

In another branch of the L-arginine metabolic pathway, we observed a decrease in the *Nos2* mRNA amount in all groups treated with rIGF-I compared with the respective control group. In the group that was not stimulated with cytokines, we observed an increase in the *Nos2* mRNA amount after siRNA transfection. Upon the addition of Th2 cytokines, an alteration in NO production was not observed (Figures 5(e) and 5(f)). Similar results were obtained using amastigotes and BALB/c peritoneal macrophages (data not shown).

### 3.6. Evaluation of the Effects of siRNA and IL-4 on IGF-I Signaling Pathways

**Figure 6:**
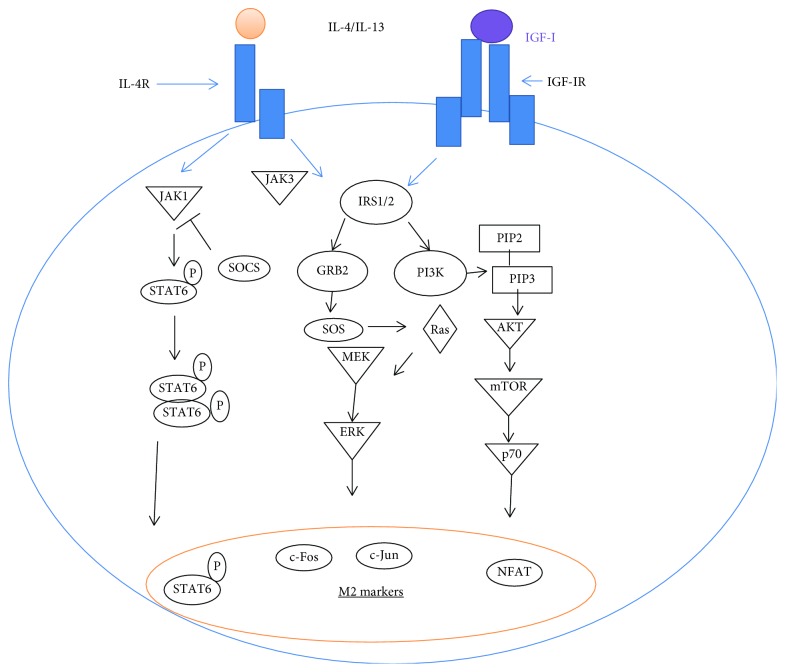
Scheme of the common components of the IGF-I and IL-4 signaling pathways.

Because our results suggest that IGF-I is necessary for IL-4 to exert its effect on parasite growth in macrophages, we examined their intracellular signaling pathways. IGF-I and the cytokines IL-4 and IL-13 share common components in their signaling pathways. IGF-I triggers MAPK (ERK) and PI3K pathways [33, 34], and IL-4 sequentially activates IRS-2 and the PI3K/Akt and Ras-MAPK pathways [35, 36] (Figure 6). The Jak-Stat pathway is also known to be triggered by IL-4.

We analyzed the key components of the IGF-I signaling pathway, that is, p44 (ERK), p38 (MAPK), and total and phosphorylated AKT, upon stimulation with IL-4 and IL-4 plus IL-13 after IGF-I silencing. We did not observe any difference in the total protein levels across all groups (data not shown); however, differences in the levels of phosphorylated proteins were observed.

**Figure 7:**
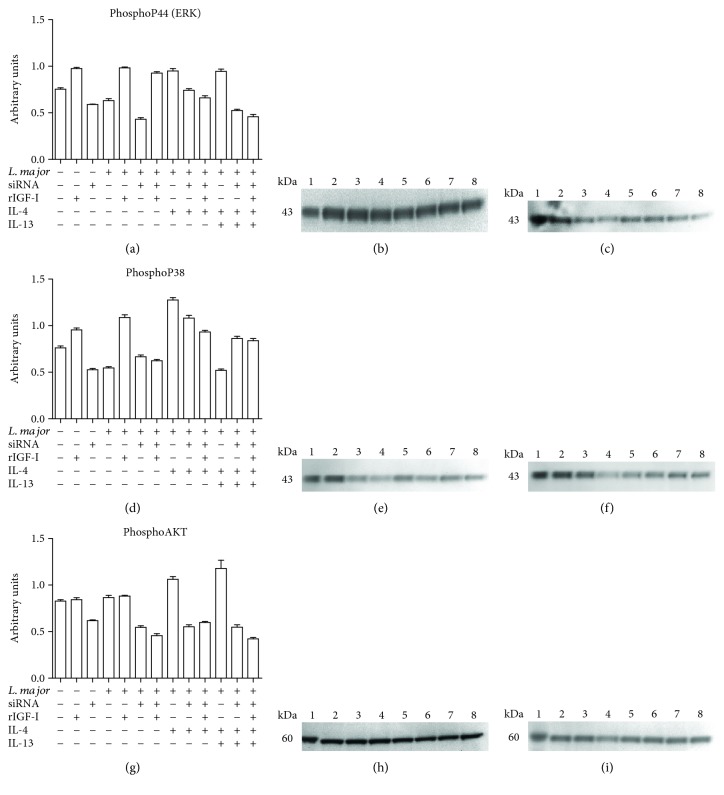
The effects of siRNA and IL-4 on components of the IGF-I signaling pathways: levels of phosphorylated p44 (ERK), p38 (MAPK), and AKT proteins. Promastigote-infected or noninfected cells transfected with or without IGF-I siRNA were stimulated for 30 minutes with IL-4 (2 ng/mL) and IL-13 (5 ng/mL). Cells were lysed, proteins were separated by 10% SDS-PAGE, and subsequently, Western blotting was performed using anti-phospho-p44 (a, b, and c), anti-phospho-p38 (d, e, and f), and anti-phospho-AKT (g, h, and i) antibodies. Protein bands corresponding to protein expression levels were subject to a densitometric analysis, and the data are expressed in arbitrary units (a, d, and g). A representative blot is shown. (b, e, h) The lanes represented the following: 1: control; 2: RAW; 3: RAW + rIGF; 4: RAW + siRNA; 5: RAW + Lm; 6: RAW + Lm + rIGF; 7: RAW + Lm + IL-4; and 8: RAW + Lm + IL-4 + IL-13. (c, f, i): 1: control; 2: RAW; 3: RAW + Lm + siRNA; 4: RAW + Lm + siRNA + rIGF; 5: RAW + Lm + siRNA + IL-4; 6: RAW + Lm + siRNA + IL-4 + rIGF; 7: RAW + Lm + siRNA + IL-4 + IL-13; and 8: RAW + Lm + siRNA + IL-4 + IL-13 + rIGF. See Materials and Methods for additional details.

Cells infected with *L. major* displayed a decrease in the levels of phospho-p44 (Figure 7(a)) and phospho-p38 (Figure 7(d)), but a difference in the phospho-AKT level was not observed (Figure 7(g)) compared with that in the noninfected RAW cells. We observed an increase in the levels of these phosphorylated proteins in all groups treated with rIGF-I compared with noninfected RAW cells. A similar increase was also observed with IL-4.

Upon silencing IGF-I expression using siRNA, all groups showed a decrease in the expression of all phosphoproteins. In IGF-I-silenced cells, Th2 cytokine stimulation did not restore the decreased expression of phospho-p44, phospho-p38, and phospho-AKT.

### 3.7. IGF-I Expression and the Effect of IGF-I on Lesion Development in *L. major*-Susceptible and -Resistant Mouse Strains

**Figure 8:**
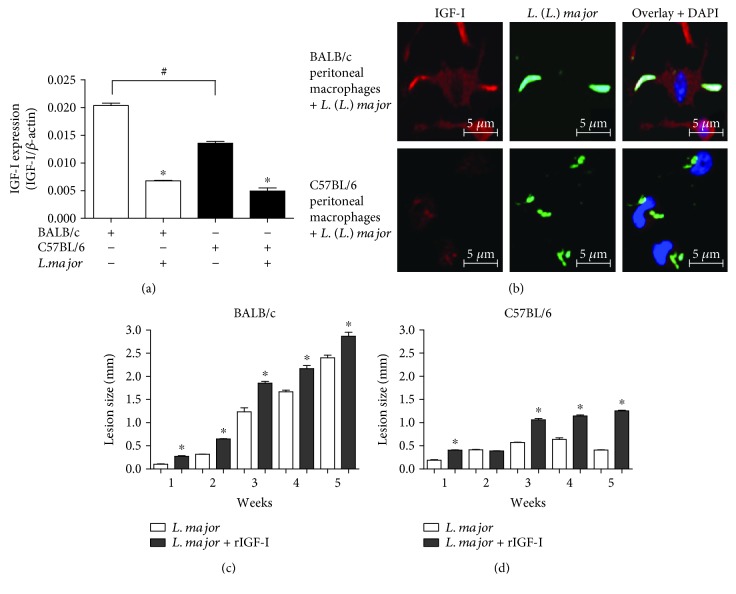
IGF-I expression and the effect of IGF-I on lesion development in *L. major*-susceptible and -resistant mouse strains. (a) The ratio of the *Igf-I* mRNA amount to *β-actin* mRNA amount in *L. major*-infected BALB/c (white bars) and C57BL/6 peritoneal macrophages (black bars) is shown. (b) Confocal microscopy was used to detect IGF-I expression via immunostaining with a 1 : 75 dilution of an anti-IGF-I antibody (using an Alexa Fluor 546-conjugated secondary antibody; red) and a 1 : 200 dilution of an anti-*Leishmania* antibody (using an Alexa Fluor 488-conjugated secondary antibody; green). DAPI (blue) was used to stain the nuclei. Images were captured using a confocal Leica LSM510 microscope with a 63x oil immersion objective. (c and d) Stationary-phase promastigotes (10^6^) that were preincubated with or without recombinant IGF-I (50 ng/mL) for 5 min were injected into the footpads of BALB/c and C57BL/6 mice, and lesion development was measured for six weeks. One representative experiment from three independent assays is shown. ^∗^*P* < 0.05 (ANOVA and Tukey's tests) compared to the respective controls. ^#^*P* < 0.05 (ANOVA and Tukey's tests) between BALB/c and C57BL/6.

We initially evaluated the differences in the expression of IGF-I to determine whether the susceptibility of BALB/c mice and the resistance of C57BL/6 mice to *L. major* infection were related to IGF-I expression. The *Igf-I* mRNA was detected in higher levels in BALB/c cells than in C57BL/6 cells (*P* < 0.05) (Figure 8(a)). Confocal microscopy indicated a correlation between the amount of *Igf-I* mRNA and IGF-I expression, confirming that the C57BL/6 peritoneal macrophages showed less IGF-I immunostaining than BALB/c cells did (Figure 8(b)).

Then, we analyzed the effect of rIGF-I on lesion development in *L. major*-infected BALB/c and C57BL/6 mice. Parasites were preincubated with 50 ng/mL IGF-I for five minutes and then washed and injected into the footpads of the mice. In control BALB/c mice, the lesions progressed continuously, but when the parasites were preincubated with IGF-I, we observed a significantly greater lesion volume than that observed in the control mice (Figure 8(c)). In control C57BL/6 mice, the lesions progressed for three weeks and then stabilized and tended to diminish, but animals infected with parasites that were preincubated with IGF-I displayed significantly greater lesions, which, interestingly, progressed continuously, similar to that in the BALB/c mice (Figure 8(d)).

## 4. Discussion

Due to the known roles of Th1 and Th2 cytokines in the resistance and susceptibility of certain inbred mouse strains to *L. major* infection and their effects on the expression of the hormone IGF-I, which has an important impact on *Leishmania* growth within host macrophages, we examined the effect of interference in the hormone IGF-I expression on the adaptive immune response. We initially analyzed the effects of Th1 and Th2 cytokines on the amount of *Igf-I* mRNA and confirmed that IGF-I expression was decreased by IFN-*γ* and increased by IL-4 and IL-13, which was consistent with previous results [4, 6]. Furthermore, when we concomitantly analyzed IGF-I expression and the parasitism of *L. major* in RAW 264.7 cells or BALB/c peritoneal macrophages *in vitro*, we observed that the macrophages stimulated with IFN-*γ* exhibited a reduction in the parasite load, accompanied by a parallel reduction in IGF-I expression and ARG1 activity and an increase in NO production. Furthermore, IL-4 and IL-13 stimulation increased parasitism, accompanied by a parallel increase in IGF-I expression and arginase activity and a reduction in NO production.

These data showing the parallel effects of those cytokines and the expression of IGF-I on parasitism compelled us to explore the interference/participation of this hormone in the actions of cytokines and other factors during *Leishmania* infection.

The idea of immune-endocrine cross talk is not new and has been described in studies examining the roles of prolactin [37], growth hormone (GH) [38, 39], IGF-I, and thyroid-stimulating hormone in the development, maintenance, and function of the immune system, which in turn cause reciprocal changes in the endocrine system [5, 40].

IGF-I exhibits pleiotropic properties, including the ability to promote cellular proliferation, differentiation, nutrient transport, energy storage, gene transcription, protein synthesis, and activation of the immune response and inflammation [12, 41]. However, its specific role in the adaptive immune response is not known. Thus, we addressed this aspect in the present study using a knockdown strategy in RAW 264.7 and mouse peritoneal cells infected with *L. major* knowing that a significant amount of IGF-I colocalizes with *L. major* in the cytoplasm of *Leishmania*-infected RAW 264.7 cells.

Using an IGF-I siRNA, we silenced the *Igf-I* mRNA in macrophages and evaluated parasitism. We observed a significant decrease in parasitism in the siRNA-transfected group compared with that in the control group without siRNA transfection in response to both promastigote and amastigote infections. This effect was reversed by the addition of rIGF-I, which induced an increase in the number of parasites, even when siRNA-mediated knockdown was maintained. This restoration observed upon the addition of recombinant IGF-I was likely induced by an increase in the levels of *Larg* mRNA and *Larg* activity, accompanied by a slight decrease in the *Nos-2* mRNA amount and NO production. Similar results were previously reported in which the addition of rIGF-I induced alternative activation of macrophages and arginase activation in *L. amazonensis*-infected macrophages [16, 17]. The present data definitively confirm the role of intrinsic IGF-I in intracellular parasite growth. Preliminary data further showed that the IGF-I siRNA decreased the parasite load in *Leishmania major*-infected BALB/c mouse footpad lesions (data not shown).

After observing such a clear-cut effect of IGF-I on parasite growth in macrophages, we proceeded to explore its role in infection development in *L. major*-infected macrophages stimulated with cytokines. After silencing *Igf-I* mRNA expression, the parasitism observed upon stimulation of the cells with cytokines did not follow the expected profile. IL-4 and IL-13 should have increased parasitism; however, they were completely ineffective when the *Igf-I* mRNA was silenced. Analyzing mRNA for components of L-arginine metabolism, we observed that the effect of IL-4 on infection progression was via the increase of *Arg1* and *Larg* mRNAs, while the effect of IL-13 on infection progression was only via the increase in *Larg* mRNA.

The effects of IL-4 and IL-13 were restored by the administration of rIGF-I to IGF-I-silenced cells in a mechanism dependent on *Leishmania* arginase production, but not by ARG1. In another study using *L. amazonensis*-infected macrophages, a novel L-arginine usage pathway independent of macrophage NOS2 and ARG1 activation was involved in the response, suggesting that *Leishmania* may have the ability to obtain access to the intracellular L-arginine pool while residing within the host macrophage [42, 43]. This evidence was confirmed in the present study, as we observed an increase in parasitism upon the addition of rIGF-I in silenced cells without changes in the amount of *Arg1* mRNA but an increase in the amount of *L*arg mRNA. Other studies have supported the importance of arginase production by the parasite since in vitro cultured *L. donovani* is capable of directly acquiring L-arginine from the medium and producing urea [44] or the deficiency in the infection and replication capabilities in *L. amazonensis* arginase knockout [45].

L-Arginine is supplied from the extracellular milieu by the cationic amino acid transporter 2 (CAT-2B), a member of the classical amino acid cationic transporter system y+ (SLC7) [42]. The observed increase in NOS2 expression may be related to an increase in CAT-2B expression since CAT-2B is required for the regulation of NOS2 activity that conversely may modulate CAT2-B expression through NO production [46–49].

The production of NO does not always follow the effect of the cytokines on parasitism, as the control of *L. major* parasitic infections does not appear to be related to NO production but rather to the presence or absence of IGF-I. Based on these results, cytokines alone are not sufficient to induce or control parasite growth, and IGF-I plays a crucial role in this process. We postulate that IGF-I may be an effector element of IL-4, which to the best of our knowledge constitutes the first evidence of cross talk between this hormone and the adaptive immune system in *L. major* infection.

Immune-endocrine cross talk involving IGF-I has been observed by others to mainly involve inflammatory cytokines IL-1 and TNF-*α*, which may inhibit both the expression of IGF-I or IGF-IR and the IGF-I-induced tyrosine phosphorylation of IRS-1 and IRS-2. This is an effect that is mediated by receptor cross talk and leads to intracellularly mediated IGF resistance [5]. Thus, these findings fundamentally differ from the findings of the present study showing that IGF-I plays a fundamental role as an effector element of Th2 cytokines.

The interaction of the endocrine and immune systems is somewhat expected, as they share several ligands and receptors in their signaling pathways. By analyzing the signaling pathways of IL-4/IL-13, which are related to the susceptibility to infection, we noticed shared components with the IGF-I pathway.

The biological effects of IGF-I occur through its binding to its receptor (IGF-IR), which is present in several cell types and tissues, mainly in macrophages. IGF-I signaling consists of two main pathways, the PI3K/Akt and mitogen-activated protein kinase (Ras/MAPK/ERK) pathways, when IRS-1 associates with the GRB2/SOS complex. IGF-I can also bind to insulin receptor (IR) when some excess of free IGF-I is present in the system that could have occurred in the present study but with lower affinity. IGF-IR and IR are highly homologous tyrosine kinase receptors sharing many common steps, inducing IRS1/2 phosphorylation and also AKT and MAPK [34, 50, 51]. Thus, if some IGF-I binds to IR, the resulted signaling will be nearly the same generating similar biological effects.

IL-4 mediates its effects through two receptors: the type I IL-4 receptor (IL-4R*α* and IL-2R*γ*) and the type II receptor (IL-4R*α* and IL-13R*α*1). Activation of these receptors results in STAT-6 phosphorylation that is necessary for the induction of the alternative macrophage pathway (M2). The ligation of the type I IL-4R activates JAK3 that participates in the activation of Akt, resulting in the induction of the M2 macrophage phenotype characterized by upregulation of molecules such as the mannose receptor, ARG1, and chitinase 3-like 3 [52]. IGF-I may also increase STAT-6 activity through IL-4, which is required to activate the expression of this cytokine [53].

In the present work, our analysis of the components of the IGF-I signaling pathway revealed increases in the amount of arginase mRNA, the phosphorylation of p44, p38, and Akt and parasitism in all groups treated either with rIGF-I or with IL-4. These results corroborated previous studies showing that IL-4 upregulates Akt activation and subsequently activates the alternative macrophage profile [54].

In the present study of *L. major* infection, we observed that all groups in which IGF-I expression was silenced using an siRNA, even IL-4-stimulated cells, showed decreases in both macrophage and parasite arginase mRNA amounts and decreases in the levels of all evaluated phosphorylated proteins. A decrease in parasitism accompanied these results, reinforcing the importance of IGF-I in the signaling induced by IL-4. In the literature, a similar study using an anti-IGF-I antibody or PI3K inhibitor in bone marrow-derived macrophages showed that IGF-I inhibition attenuated the IL-4-induced increases in the mRNA amount of M2 markers, such as mannose receptor, *Arg1* mRNA and chitinase 3-like 3, as well as ARG1 protein expression, suggesting that IGF-I is required for IL-4-induced Akt phosphorylation and M2 activation [52]. Based on this evidence, we postulate that IGF-I is the element that actually activates the PI3K/Akt pathway, which IL-4 uses. In IFN-*γ*-treated cells, the application of the IGF-I siRNA strengthened the effect of decreasing the parasite load, and the addition of rIGF-I almost nullified this effect, returning it to levels similar to the control group. Reinforcing our finding, the inhibition of PI3K or treatment with an anti-IGF-I antibody exacerbates the effects of IFN-*γ* [52].

Our data are original in that they clearly show the interference of IGF-I on the IL-4 effect. In experimental visceral leishmaniasis, IGF-I increases the expression of arginase, while the deletion of IGF-IR leads to decreased arginase in a STAT-6-dependent mechanism and restriction of *L. donovani* growth [55]. These authors explored the role of extrinsic IGF-I but not of intrinsic IGF-I present within the cell, and they did not explore the interference of IGF-I on the IL-4 effect. Furthermore, it is known that the immune responses to *Leishmania* infection vary considerably according to the species of the parasite. In particular, the immune response to *L. donovani* and *L. infantum*, which are strains leading to visceral leishmaniasis, is quite different from the immune response established in the *L. major* model. In experimental visceral leishmaniasis, the role of IL-4 in the susceptibility to visceral disease is not clearly defined [55, 56]. Moreover, L-arginine metabolism of *L. donovani* is shown to be different from that of other species [57].

Regarding *Leishmania* infections in experimental models, we hypothesized that the profiles of mouse resistance and susceptibility to *L. major* infection are not only due to the effects of Th1 and Th2 cytokines, but that IGF-I may also play an important role in infection development due to constitutive differences in IGF-I expression. The BALB/c and C57BL/6 mouse strains did not exhibit similar expression profiles of IGF-I, with the latter displaying lower expression. Similarly, lower serum IGF-I levels have been detected in C57BL/6 mice compared to that in C3H/HeJ mice [29]. To address this issue, we analyzed the effect of IGF-I in vivo and found that lesion development in mice was increased when *L. major* promastigotes were preincubated with recombinant IGF-I and applied to susceptible and resistant mouse strains. The lesions in BALB/c mice increased in size and were progressive, but the profile of lesion development was more striking in C57BL/6 mice, as the lesions in mice injected with IGF-I-preincubated parasites not only increased in size but also became progressive, even in the late phase when the lesions in the control animals were diminishing. This resistant-to-susceptible profile reversion was not observed in certain previous studies [25, 28]. Although the lesions in C57BL/6 mice increased progressively following treatment with rIGF-I, the lesion size was smaller than lesions in BALB/c mice. This difference might be due to the IL-4 production pattern in these mouse strains. IL-4 production is similar in the initial phase of infection in both susceptible and resistant mice; however, the production of this cytokine in resistant mice is transient [26]. Another study also showed a nonsustained IL-4 level. In resistant mice, treatment with rIL-4 at the beginning of the infection did not induce the susceptible profile due to the noncontinuous production of IL-4 during the infection [25]. Thus, the main characteristics and differences in terms of susceptibility and resistance observed in certain *L. major*-infected mouse strains may be due to cytokines to some extent, but the susceptibility essentially depends on the presence of IGF-I.

## 5. Conclusions

The present study provides new insights into the immunology of leishmaniasis, reinforcing the importance of IGF-I in *Leishmania* infection by revealing a significant immune-endocrine interaction in this context. Our data strongly suggest that IGF-I is an effector element of IL-4 actions, leading to M2 macrophage differentiation, involving the PI3K/Akt pathway during *L. major* infection.

Our findings raise questions about pathogenic processes in other diseases in which Th2 cytokines play an important role. In relation to the pathogenesis of leishmaniasis, a polymorphism in the IGF-I gene has been detected in the human population [58, 59]; thus, we speculate that individuals in endemic areas will be susceptible or resistant or will exhibit exacerbation or modulation of the pathogenic process, depending on the expression of basal IGF-I.

## Data Availability

The data used to support the findings of this study are available from the corresponding author upon request.
